# Ultra-processed food intake is associated with increased gastrointestinal tract symptoms and alterations in gut microbiota in patients with systemic sclerosis

**DOI:** 10.3389/fimmu.2025.1610360

**Published:** 2025-10-09

**Authors:** Ju Young Lee, Swapna Mahurkar-Joshi, Arissa Young, Jennifer S. Labus, Bofei He, Ezinne Aja, Jonathan P. Jacobs, Elizabeth R. Volkmann

**Affiliations:** ^1^ David Geffen School of Medicine, University of California, Los Angeles, Los Angeles, CA, United States; ^2^ G. Oppenheimer Center for Neurobiology of Stress and Resilience, David Geffen School of Medicine, University of California, Los Angeles, Los Angeles, CA, United States; ^3^ Goodman-Luskin Microbiome Center, University of California, Los Angeles, Los Angeles, CA, United States; ^4^ The Vatche and Tamar Manoukian Division of Digestive Diseases, David Geffen School of Medicine, University of California, Los Angeles, Los Angeles, CA, United States; ^5^ Division of Rheumatology, Department of Medicine, David Geffen School of Medicine, University of California, Los Angeles, Los Angeles, CA, United States

**Keywords:** systemic sclerosis, microbiome, ultra processed food, scleroderma, gut microbiome

## Abstract

**Background:**

Alterations in the gastrointestinal (GI) microbiome (i.e., dysbiosis) are a feature of systemic sclerosis (SSc). Diet is a known modifier of the GI microbiome, and ultra-processed food (UPF) consumption has been associated with adverse changes in GI microbial composition. This study aimed to determine whether UPF consumption affects the GI microbiota and GI symptoms in patients with SSc.

**Methods:**

Adult SSc patients provided stool samples and completed both the Diet History Questionnaire II (DHQ-2) and the UCLA Scleroderma Clinical Trial Consortium Gastrointestinal Tract Instrument (GIT 2.0). Shotgun metagenomics were performed using the Illumina NovaSeq 6000 with a target depth of 10 million 150x2 sequences per sample. UPF items (N=54) on the DHQ-2 were identified using the NOVA scale of food classification, and UPF intake was calculated as gram-per-week consumption according to patient reported frequency. General linear models were created to identify differentially abundant species based on UPF consumption and to evaluate the relationship between UPF consumption and GI symptoms as measured by the GIT 2.0. These models adjusted for body mass index (BMI), current proton pump inhibitor (PPI) use, current probiotic use, current or prior immunomodulatory therapy, and presence of small intestinal bacterial overgrowth (SIBO).

**Results:**

Of the 65 total SSc patients included, 84.6% were female. The mean age was 53.83 ± 13.19 years, and the mean BMI was 25.25 ± 4.75. The median UPF consumption was 2395.82 g/week. Increased UPF consumption was significantly associated with increased GI symptoms in our multivariate model (β=0.34; p<0.01). Among 257 species analyzed, 5 bacterial species were significantly associated with UPF consumption in the multivariate models, including *Limosilactobacillus* fermentum (β=0.32; p<0.01) and *Faecalicatena fissicatena* (β= -0.36; p-value<0.01), while the abundance of 6 bacterial species was significantly associated with GI symptom severity after adjusting for the aforementioned covariates.

**Conclusions:**

SSc patients reporting a higher UPF consumption demonstrated alterations in GI microbial composition as well as increased GI symptoms, even after adjusting for factors known to affect the microbiota of patients with SSc. Future studies are needed to determine whether interventions aimed at lowering UPF consumption may improve GI outcomes for patients with SSc.

## 1 Introduction

Systemic sclerosis (SSc) is a complex autoimmune disorder characterized by chronic inflammation and fibrosis across multiple organ systems, with the gastrointestinal tract being the most common internal organ involved (1). Studies show that up to 90% of patients with SSc experience GI dysfunction, most frequently in the form of esophageal disease (2, 3). While lower GI tract involvement, such as gastroparesis, constipation, or fecal incontinence, is less common, these symptoms not only contribute to malnutrition and adverse health outcomes, but also significantly impact quality of life and emotional well-being (4, 5).

The pathophysiology underlying lower GI tract dysfunction in SSc is poorly understood. However, emerging evidence points to the gut microbiome as a key contributor. Specifically, unique alterations in the gut microbiota (i.e., dysbiosis), have been observed in patients with early SSc that suggest dysbiosis may be a contributing factor to disease progression (6). Moreover, specific SSc disease manifestations, such as interstitial lung disease (ILD) and small intestinal bacterial overgrowth (SIBO), are associated with unique microbial profiles in SSc (7). These changes, which involve a reduction in species diversity with an overrepresentation of pathobiont species, have also been observed in other conditions associated with systemic chronic inflammation, such as systemic lupus erythematosus (SLE), inflammatory bowel disease (IBD), and type 2 diabetes (8–11). The shift in favor of pathobionts is thought to trigger increases in intestinal permeability, immune dysregulation, and systemic inflammation; however, the factors driving these microbial alterations are likely multifactorial (12, 13).

Diet is a known modifier of the microbiome, and changes in diet can induce microbial shifts within days (14, 15). Consumption of ultra-processed food (UPF) (i.e., industrial formulations made entirely or mostly from food extracts, derivatives, and additives, designed to be hyper-palatable) specifically has been associated with dysbiosis and decreased bacterial diversity (16–18). Increased UPF consumption has been linked to a host of chronic illnesses, including metabolic syndrome, cardiovascular disease, cancers, depression, IBD, and renal function decline (19–23).

Given the increasing reliance of UPFs in the Western diet, understanding their potential consequences on the microbiome and human health and diseases is a growing area of interest. However, no prior studies have investigated the impact of UPF consumption on GI symptoms and the GI microbiome and in patients living with SSc. The present study aimed to test the following hypotheses: (1) increased UPF consumption is associated with increased GI symptoms in patients with SSc; (2) alterations in species abundance are associated with increased UPF consumption and GI symptoms severity. In exploratory analyses aimed at generating hypotheses for future studies we investigated whether the UPF consumption mediates the association between the gut microbiome and GI symptom severity. The findings of this research may inform the development of evidence-based nutritional guidelines for the management of GI disease in SSc in the future.

## 2 Materials and methods

### 2.1 Study participants

Participants were patients who were consecutively recruited from the University of California, Los Angeles (UCLA) Scleroderma Center clinic from January 2014 to November 2022. Inclusion criteria were adult patients (age ≥18 years) with SSc of any disease duration according to the 2013 American College of Rheumatology/European League Against Rheumatism Classification Criteria for SSc (24). Exclusion criteria included the presence of a comorbid GI condition such as IBD, GI malignancy, or celiac disease. Participants were also excluded if they were unable to refrain from using antibiotics for a minimum of three weeks prior to stool collection or had taken more than 2 courses of antibiotics in the preceding year. Patients could continue the use of antacids, histamine H2-receptor antagonists, immunomodulatory drugs, and proton pump inhibitors (PPIs) to minimize unnecessary morbidity, but were required to discontinue laxatives, promotility agents, and anti-diarrheal medications at least one week prior to stool collection.

Clinical features of the participants were obtained via chart review (**Table 1**). SSc disease duration was based on the time from onset of first non-Raynaud symptom to the date of stool collection. Medication history was self-reported by the participant and independently verified by the study team using the electronic medical record. Immunomodulatory use was defined as any use of immunomodulatory drugs from disease onset to the date of stool collection. The presence of ILD was determined by high resolution computed tomography of the chest (HRCT). The presence of other disease features, such as SIBO, were determined based on physician-based diagnoses.

**Table 1:** Baseline characteristics of SSc cohort based on UPF consumption.

	All participants (N=65) (N=65)	Low consumption (N=33) (N=33)	High consumption (N=32) (N=32)	t-value (High *vs* Low consumption)	p-value (t-test or Fisher’s Exact test^#^)
Age (years), Mean (SD)	53.83 (13.19)	51.69 (14.87)	55.91 (11.17)	1.291	0.202
BMI, Mean (SD)	25.25 (4.75)	25.56 (4.91)	24.94 (4.64)	-0.521	0.604
Female, N (%)	55/65 (84.6)	29/33 (87.9)	26/32 (81.3)	0.16	0.511^#^
Race, N (%)					0.077^#^
White	46/65 (70.8)	20/33 (60.6)	26/32 (81.3)		
Black	1/65 (1.5)	1/33 (3.0)	0/32 (0.0)		
Asian	8/65 (12.3)	7/33 (21.2)	1/32 (3.1)		
Other1	10/65 (15.4)	5/33 (15.2)	5/32 (15.6)		
UPF intake, g/week, Mean (SD)	2782.44 (2182.07)	1229.08 (596.05)	4384.34 (2060.55)	8.33	6.40E-10
GIT 2.0 score, Mean (SD)	0.536 (0.544)	0.450 (0.419)	0.628 (0.646)	1.28	0.207
Disease duration, years, Median (Range)	7.16 (0.41–44.34)	6.13 (0.86–34.33)	8.43 (0.41–44.37)	0.58	0.562
Diffuse cutaneous SSc, N (%)	32/64 (50.0)	16/33 (48.4)	16/31 (51.6)		1.000^#^
Anti-Centromere positive, N (%)	15/55 (27.3)	6/29 (20.7)	9/26 (34.6)		0.462^#^
Anti-Scl-70 positive, N (%)	22/59 (37.3)	13/29 (44.8)	9/30 (30.0)		0.391^#^
Anti-RNAP III positive, N (%)	6/28 (21.4)	4/18 (22.2)	2/10 (20.0)		0.155^#^
Presence of SIBO, N (%)	19/65 (29.2)	9/33 (27.2)	10/32 (31.3)		0.789^#^
Presence of ILD, N (%)	36/65 (55.3)	20/33 (60.6)	16/32 (50.0)		0.459^#^
Current PPI use, N (%)	51/65 (78.5)	27/33 (81.8)	24/32 (75.0)		0.558^#^
Probiotic use, N (%)	17/65 (26.1)	11/33 (33.3)	6/32 (18.8)		0.260^#^
Current or prior immunosuppression, N (%)	55/65 (84.6)	27/33 (81.8)	28/32 (87.5)		0.733^#^
Any alcohol use, N (%)	22/64 (34.4)	9/32 (28.1)	13/32 (40.6)		0.363^#^

^1^Including those who identified as more than one race, Native Hawaiian or other Pacific Islander, or those whose race was unknown. ^#^, p-values from Fisher’s exact tests.

The UCLA Institutional Review Board (#13-001089) approved the study protocol and written informed consent was obtained from each participant.

### 2.2 Sample collection, gene sequencing, and microbiome analysis

Patients provided stool samples using a previously published home collection method (25). Samples were frozen and stored at -80°C while awaiting processing and analysis. Shotgun metagenomics were performed using the Illumina NovaSeq 6000 with a target depth of 10 million 150x2 sequences per sample. Shotgun reads were inputted into MetaPhIAn4 for taxonomic identification of species for compositional analysis and subsequently underwent center log-ratio transformation (26). Samples were filtered to retain species with at least 10% non-zero counts resulting in 257 species for the final analyses.

### 2.3 Assessment of UPF consumption and GI symptoms

#### 2.3.1 UPF consumption

On the day of the stool collection, all participants completed the Diet History Questionnaire II (DHQ-2), a valid, 142-item food frequency questionnaire that assesses the intake of specific foods, beverages and condiment items consumed during the past four weeks (27). For each item, patients reported the portion size and frequency of consumption. Intake of each item was then calculated by multiplying the typical portion size of the item in grams by its reported frequency of consumption in weeks to create a continuous variable of gram-per-week. The weight of each portion per item was taken from the associated Nutrient Database from the National Cancer Institute (28). Using the NOVA scale of food classification, 54 items on this questionnaire were identified as being ultra-processed, or Group 4 (29, 30) (**Supplementary Table S1**). Select foods (N=17) for which portion size was unable to be calculated due to ambiguity such as margarine and cream cheese were excluded. The sum of these items was calculated to determine the participant’s total UPF consumption in gram-per-week. The participants were then grouped into low or high UPF consumption based on the median UPF consumption value.

#### 2.3.2 GI symptom severity

To assess GI symptom severity, all participants also completed the UCLA Scleroderma Clinical Trial Consortium Gastrointestinal Tract Instrument (GIT 2.0) on day of stool collection. The GIT 2.0 is a self-reported questionnaire that measures GI symptom severity and quality of life in patients with SSc. It consists of 34 items across 7 scales (reflux, distention/bloating, fecal incontinence, diarrhea, social functioning, emotional well-being, and constipation) (31). The total score averages 6 of the 7 scales and is scored from 0-3, with higher scores indicating worse SSc-GI symptoms and GI-related quality of life.

### 2.4 Statistical analysis

Participant characteristics were summarized using means and standard deviations for continuous variables and frequencies and percentages for categorical data. Furthermore, independent t-tests and Fisher’s exact tests were applied to test for differences in these characteristics between low and high UPF subgroups based on the median. The Shannon diversity index was used to compare species richness and evenness of microbial communities between the low and high UPF subgroups, while robust principal component analysis (rPCA) was used to compare global compositional differences (i.e., beta diversity) between the low and high UPF subgroups.

#### 2.4.1 Association analyses

The associations between each of the 257 species abundances with GI symptom severity and UPF consumption (measured continuously) were tested using general linear models (GLMs), controlling for factors known to affect intestinal microbial composition in prior studies. These factors were determined *a priori* and included body mass index (BMI), current PPI use, current supplemental probiotic use, current or prior immunomodulatory therapy, and presence of SIBO. We considered a p<0.05 as the threshold for reporting. We report the unstandardized beta estimates (b), standard error, and standardized betas (β) as a measure of effect size estimates (32).

#### 2.4.2 Mediation analyses

To examine the mediating (indirect) effect of UPF consumption on the association between the predictor variable (i.e., specific bacterial species), and the outcome variable (i.e., GI symptom severity), we applied exploratory mediation analyses using simultaneous linear regression equations implemented via structural equation modeling (33–35). Bacterial species whose abundance was significantly associated (p<0.05) with GI symptom severity based on the above analyses were included in the mediation analysis. All the models adjusted for the aforementioned covariates (e.g., BMI, current PPI use, current supplemental probiotic use, current or prior immunomodulatory therapy, and presence of SIBO). The significance level was set at p<0.05. Standardized β and confidence intervals for indirect effects were reported. All analyses were performed using R (36). Mediation analyses were performed using the *lavaan* (37). The bootstrapped 95% percentile confidence intervals for the indirect effect were obtain using the function *standardizedSolution_boot_ci()* from the package *semhelpinghands* (38, 39). For indirect effects, confidence intervals that do not contain zero suggest statistically significant mediation.

## 3 Results

### 3.1 Participant characteristics

Among the 65 SSc patients included in the study, the majority were female (84.6%) (**Table 1**). The mean age was 53.83 ± 13.19 years, and the mean BMI was 25.25 ± 4.75. The median UPF consumption was 2395.82 g/week, while the mean UPF consumption was 2782.44 g/week with a range of 174.55–9231.91 g/week (SD=2165.22). The mean GIT 2.0 score for the cohort was 0.55 (SD=0.56). The median disease duration was 7.16 years, with a range of 0.41–44.34 years. ILD was observed on HRCT in 55.3% (N=36) of participants, and 29.2% (N=19) had SIBO. Most patients (84.6%; N=55) reported current or prior use of immunomodulatory medication.

No statistically significant differences were observed in patient characteristics between the low and high UPF consumption cohorts (**Table 1**). Moreover, no statistically significant differences were observed in alpha diversity (Kruskal-Wallis H=0.45; p=0.50) or beta diversity (p=0.90) between the two UPF subgroups.

### 3.2 Higher UPF consumption is associated with worse GI symptoms

**Figure 1:**
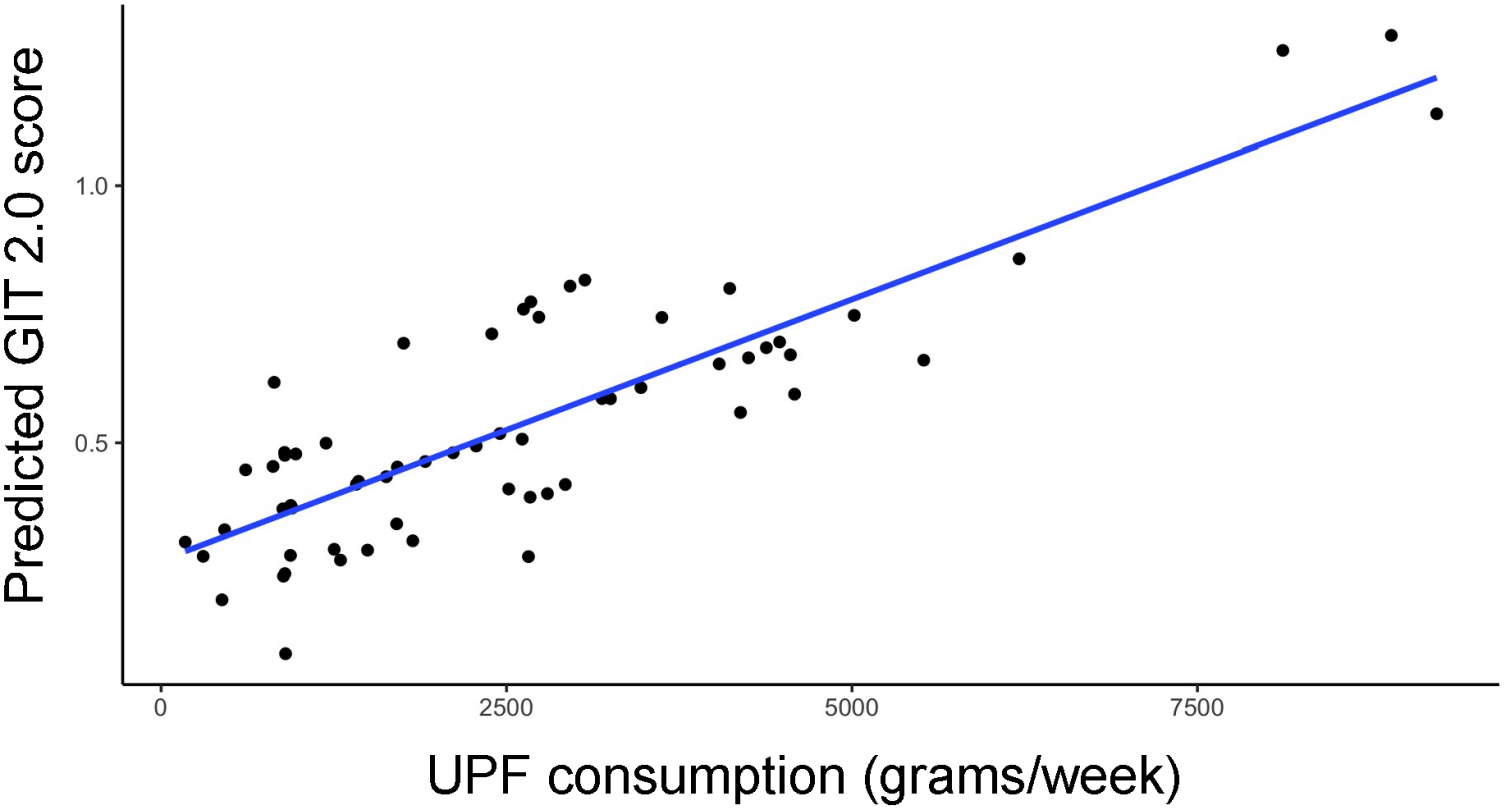
Relationship between predicted GIT 2.0 score (y-axis) and UPF consumption (x-axis, grams/week) based on generalized linear model estimates, adjusting for BMI, PPI use, probiotic use, immunomodulatory use, and presence of SIBO.

In the entire cohort, increased UPF consumption was significantly associated with increased GI symptoms in our multivariate model (β=0.34; p<0.01) (**Figure 1**).

### 3.3 Specific bacterial species are associated with UPF consumption

**Figure 2:**
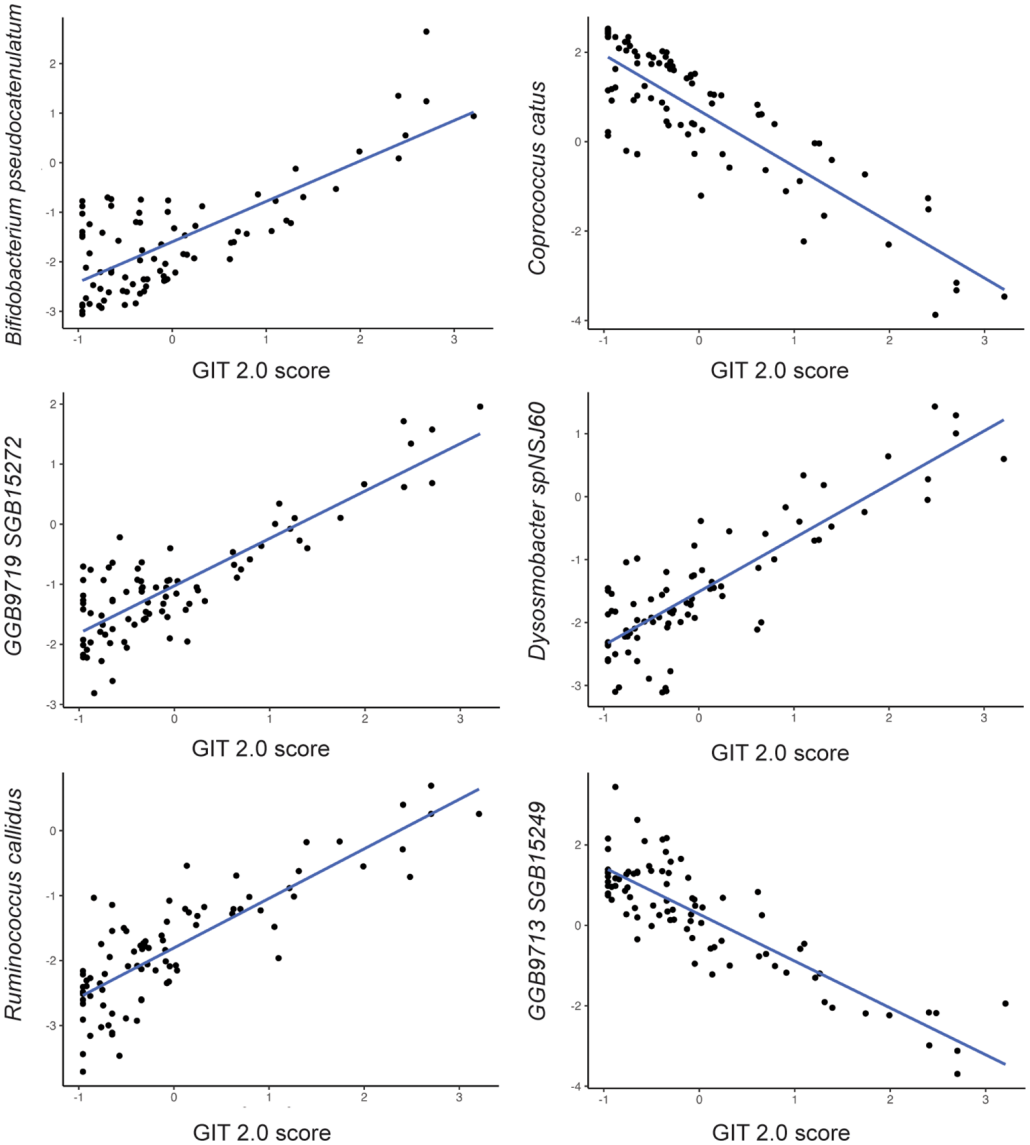
Relationship between bacterial species abundance (y-axis) and GIT 2.0 score (x-axis) based on generalized linear model estimates, adjusting for BMI, PPI use, probiotic use, immunomodulatory use, and presence of SIBO.

**Figure 3:**
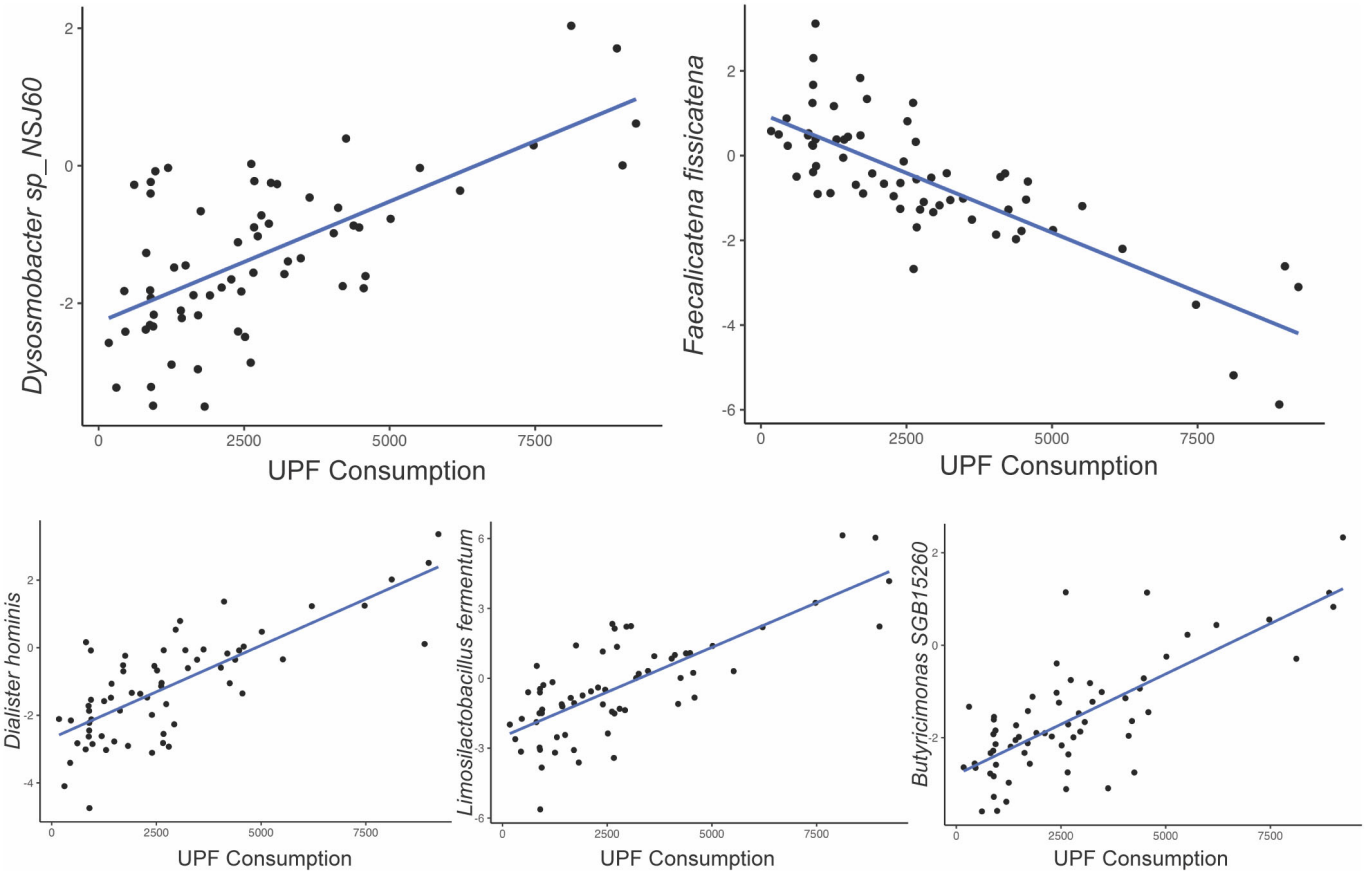
Relationship between bacterial species abundance (y-axis) and UPF consumption (x-axis, grams/week) based on generalized linear model estimates, adjusting for BMI, PPI use, probiotic use, immunomodulatory use, and presence of SIBO.

Among the 257 species identified, the abundance of 10 bacterial species was significantly altered based on UPF consumption. After adjusting for covariates, 5 bacterial species remained significantly associated with UPF consumption (**Table 2**; **Figure 2**). Increased UPF consumption was associated with increased abundance of *Butyricimonas SGB15260* (β=0.29; p-value<0.02); *Limosilactobacillus fermentum* (β=0.32; p-value<0.01); *Dysosmobacter NSJ60* (β=0.28; p-value<0.03); and *Dialister hominis* (β=0.26; p-value<0.04). By contrast, increased UPF consumption was associated with decreased abundance of *Faecalicatena fissicatena* (β= -0.36; p-value<0.01) (**Figure 3**).

**Table 2:** Bacterial species significantly associated with UPF consumption and/or GI symptom severity after adjusting for BMI, PPI use, probiotic use, SIBO, and use of immunomodulatory therapies.

Species	Family	b	SE	β	*p*-value	*q-value*
UPF consumption						
*Butyricimonas_SGB15260*	Odoribacteraceae	4.1E-4	1.79E-4	0.29	0.02	0.866
*Limosilactobacillus fermentum*	Lactobacillaceae	7.24E-4	2.75E-4	0.32	0.01	0.866
*Faecalicatena fissicatena*	Lachnospiraceae	-5.94E-4	2.07E-4	-0.36	0.01	0.866
*Dysosmobacter_sp_NSJ_60*	Ruminococcaceae	4.02E-4	1.86E-4	0.28	0.03	0.866
*Dialister hominis*	Veillonellaceae	4.30E-4	2.08E-4	0.26	0.04	0.866
GI symptom severity						
*Bifidobacterium pseudocatenulatum*	Odoribacteraceae	0.86	0.42	0.23	0.04	0.944
*Coprococcus catus*	Lactobacillaceae	-1.13	0.46	-0.28	0.02	0.944
*GGB9713_SGB15249*	Lachnospiraceae	0.76	0.33	-0.28	0.02	0.944
*Dysosmobacter_sp_NSJ_60*	Ruminococcaceae	-1.07	0.44	0.26	0.02	0.944
*GGB9719_SGB15272*	Veillonellaceae	0.87	0.43	0.23	0.05	0.944
*Ruminococcus callidus*	Ruminococcaceae	0.72	0.34	0.25	0.03	0.944

b, unstandardized beta; SE, standardized error; β, standardized-beta.

### 3.4 Specific bacterial species are associated with GI symptom severity

Among the 257 species identified, the abundance of 6 bacterial species was significantly associated with GI symptom severity after adjusting for the aforementioned covariates (**Table 2**). For example, the abundance of *Bifidobacterium pseudocatenulatum* (β=0.23; p-value<0.04); *Ruminococcus callidus* (β=0.25; p-value<0.03); *GGB9719 SGB15272* (β=0.23; p-value<0.05); *Dysosmobacter* sp*NSJ60* (β=0.26; p-value<0.02) was positively associated with GI symptom severity; whereas the abundance of *Coprococcus catus* (β= -0.28; p-value<0.02) and *GGB9713 SGB15249* (β= -0.28; p-value<0.02) was negatively associated with GI symptom severity. Notably, *Dysosmobacter NSJ60* was significantly associated with both UPF intake and GIT 2.0 scores.

### 3.5 Does UPF consumption mediates the relationship GI microbial species and GI symptoms?

**Figure 4:**
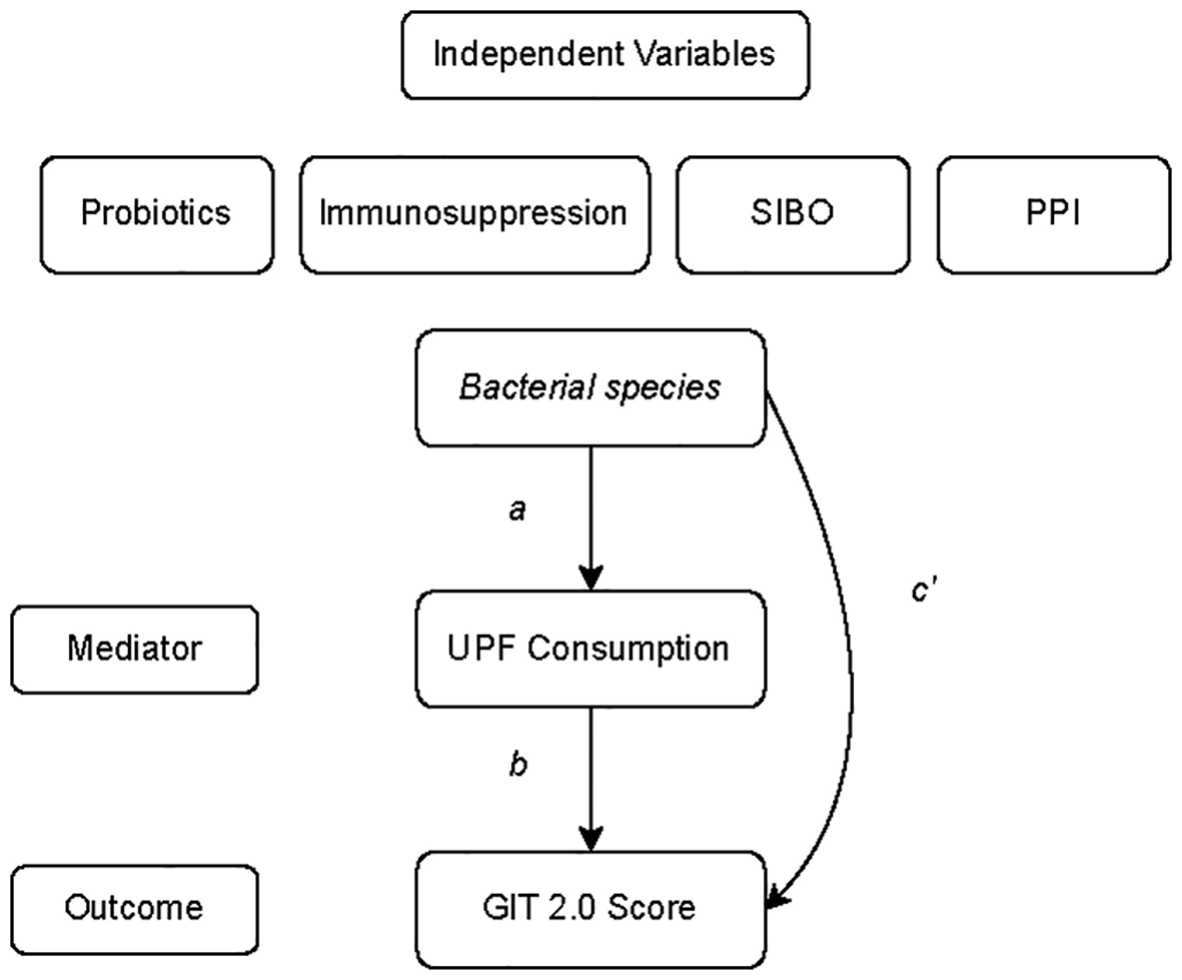
General model of the mediation analysis describing the relationship between bacterial species whose abundance was significantly associated with GI symptom severity or UPF consumption (predictor variable), UPF consumption (mediator), and GIT 2.0 score (outcome). Individual paths are denoted as a, b, or c’.

Next, we tested whether UPF consumption mediates the influence of specific gut microbial species (predictors) on GI symptom severity (outcome) via mediation analysis (**Figure 4**). In this analysis, the 6 bacterial species identified as being significantly associated with GI symptoms were entered into the models as predictors. While the mediation analysis revealed several direct effects, there was no evidence that UPF consumption statistically mediated the observed associations between the species and GI symptom scores (**Table 3**). In other words, the relationship between GI species and GI symptoms did not appear to be mediated by UPF intake, even though increased UPF intake was associated with worse GI symptoms.

**Table 3:** Estimated standardized beta coefficients from the mediation analyses describing the relationship between bacterial species (independent variables), UPF consumption (mediating variable), and GIT scores (outcome).

Species	Species→UPF (a) β [95% CI]	UPF→GIT (b)β [95% CI]	Species→GIT (c’) β [95% CI],	Indirect effect (a*b)
*Dysosmobacter_sp_NSJ_60*	0.314* [0.08; 0.547]	0.239* [0.004; 0.474]	0.283* [0.051; 0.515]	0.075 [-0.058; 0.197]
*Bifidobacterium pseudocatenulatum*	0.035 [-0.211; 0.28]	0.321* [0.096; 0.547]	0.234* [0.012; 0.456]	0.011 [-0.064; 0.084]
*Coprococcus catus*	0.037 [-0.216; 0.291]	0.341* [0.121; 0.561]	-0.316 [-0.54; -0.092]	0.013 [-0.077; 0.102]
*GGB9713_SGB15249*	0.04 [-0.209; 0.288]	0.34* [0.116; 0.564]	-0.252* [-0.476; -0.028]	0.013 [-0.095; 0.115]
*GGB9719_SGB15272*	0.086 [-0.155; 0.327]	0.306* [0.082; 0.53]	0.256* [0.039; 0.474]	0.026 [-0.05; 0.113]
*Ruminococcus callidus*	0.146 [-0.094; 0.387]	0.302* [0.071; 0.533]	0.182* [-0.044; 0.407]	0.044 [-0.029; 0.194]

Individual paths are denoted as a, b, or c’. Statistical significance (p<0.05) is denoted by * after coefficient.

## 4 Discussion

GI involvement occurs in most patients with SSc, and currently no disease modifying therapies are available to prevent progression of this troubling manifestation of SSc. Patients often make dietary modifications to try to ameliorate their GI symptoms; however, nutritional studies in this disease are sparse, and currently, no evidence-based recommendations regarding diet in SSc exist. The present study is the first to demonstrate a relationship between UPF consumption and GI symptoms in SSc. First, we discovered that SSc patients with higher UPF consumption reported increased GI symptoms compared to those with lower UPF consumption. Second, we found that specific bacterial species were associated with UPF consumption and GI symptoms. Finally, we found that UPF consumption and GI symptoms are directly linked suggesting that factors outside of the GI microbiome may also influence this relationship. Taken together, the findings of this study suggests that lowering UPF consumption may potentially help to GI symptoms in patients with SSc.

The mean UPF consumption in the present SSc cohort was 2782.44 g/week, which is similar to data reported from other studies in developed nations, including studies conducted in Brazil (approximately 2562 g/week) and Spain (approximately 2692 g/week) (20, 40). However, the observed UPF consumption in the present cohort is increased compared to a study conducted in Quebec, Canda (approximately 1575 g/week) (41). Among those patients in the high UPF consumption group in the present SSc cohort, the mean intake was 4384.34 g/week. This is again comparable to the aforementioned Brazilian and Spanish cohorts, in which the mean intake in the high UPF consumption groups was 5145 g/week and 4448 g/week, respectively (20, 40).

Increased UPF consumption was also significantly associated with increased GI symptoms in our multivariate model. These findings are consistent with studies in other GI diseases, including irritable bowel syndrome (IBS) and functional constipation (42, 43). The potential mechanism by which UPFs affect GI symptoms are not completely understood. However, the low fiber content of UPF foods could potentially worsen symptoms, such as constipation. In addition, studies have demonstrated that additives found in UPFs, such as artificial sweeteners and emulsifiers, can impact the integrity of the intestinal barrier leading to increased intestinal permeability, apoptosis of intestinal epithelial cells, and impaired mucus production, all of which can contribute to bacterial translocation and increased local and systemic inflammation (44).

In the setting of SSc, a disease state associated with dysbiosis, increased UPF consumption likely further exacerbates alterations in the composition of the GI microbiome. Cueva-Sierra and colleagues (18) demonstrated decreased alpha-diversity in men who consumed higher quantities of UPFs, and emerging studies report decreased bacterial diversity following exposure to UPF ingredients like artificial sweeteners and emulsifiers (44). In the present study, we did not detect significant differences in alpha or beta diversity between the high and low UPF subgroups; however, we dichotomized subgroups based on the median. Since there is no valid definition of “high UPF” intake, it is possible that our dichotomization procedure masked associations that may have been apparent when a different threshold was used to define subgroups. The study by Cueva-Sierra and colleagues (18) defined high UPF intake as 5 or more servings per day. Since our study captured UPF intake over the prior four weeks, we were not able to validate their approach in the present manuscript.

However, we did detect differences at the species level in our multivariate models that included UPF intake as a continuously measured variable. For example, we found that increased relative abundance of *Limosilactobacillus* (previously named *Lactobacillus*) *fermentum* was associated with high UPF intake. This may be in part due to our study population, as prior studies have demonstrated increased abundance of the commensal *Limosilactobacillus* in SSc patients compared with healthy controls (6, 45).

*Bifidobacterium pseudocatenulatum and Ruminococcus callidus* were also positively associated with GI symptom severity. The abundance of the genus *Bifidobacterium* is increased in prior studies investigating the microbiota of SSc patients, and it is also increased in women and those with high UPF diets (6, 18, 45, 46). *Ruminococcus*, a genera traditionally deemed pathobiont, was increased in abundance in prior studies of SSc patients (6, 47). It is important to note that this is the first study to report species-level associations with GI symptoms in SSc, as prior studies investigated only genus-level associations. Therefore, these associations require replication in other cohorts.

While our mediation analysis did not demonstrate that UPF consumption mediated the relationship between species abundance and GI symptoms, it is conceivable that our study was underpowered to detect significant indirect effects in this exploratory analysis. It is also possible that our mediation analysis did not account for all possible confounding factors, although we did attempt to adjust for those variables consistently found to affect GI microbial composition in prior studies.

Our study should be considered under the context of certain limitations. First, the study is cross-sectional and therefore, the relationships observed may not be causational or persist with time. Future prospective studies are needed; specifically, controlled studies that assess the impact of limiting UPF intake on progression of SSc-GI symptoms over time. Second, this is a single-center study and as such, the results may not be generalizable to other populations of SSc patients, particularly those patients who reside in countries where UPF consumption is limited. Third, our sample size is relatively small; however, we observed significant associations that are consistent with those reported in prior studies, suggesting that the findings are unlikely to be due to chance alone. Fourth, the questionnaires used to assess UPF intake are based on patient report and likely suffer from recall bias. Moreover, we had to exclude certain foods that could be considered UPF when their exact quantity of UPF could not be estimated. Future prospective studies that use a daily electronic food diary could minimize the risk of recall bias.

The present study has important strengths. First, it is the first study to use shotgun metagenomics to obtain species-level resolution of the GI microbiome in patients with SSc and investigate associations between species abundance and GI symptoms. Prior microbiome studies in SSc have mainly utilized 16S sequencing, which yielded microbiota data limited to the genus level. Given the fact that some species within in a genus can act as commensal organisms and others as pathobionts, our study represents an important advance in this field. Second, we adjusted our analyses for potential confounders known to affect the colonic microbiota, including probiotics and immunomodulatory medications. Lastly, we ensured our patients withheld medications, such as antibiotics, at least 3 weeks prior to stool collection via thrice-verification of patient medication lists.

In summary, this is the first study to report the relationship between UPF consumption and GI symptoms in patients with SSc. The findings demonstrate that the relationship between UPF intake and GI symptoms is moderated in part by alterations in the GI microbiome. The results also suggest that minimizing UPF consumption could potentially improve GI symptoms in patients with SSc, although future prospective, controlled trials are needed to test this hypothesis. Given the multitude of adverse health outcomes linked to UPF intake in the general population, limiting the intake of UPF has the potential to improve quality of life for patients living with SSc.

## Data Availability

The datasets presented in this article are not readily available because ethics approval was not obtained by all participants to publicly share data. Requests to access the datasets should be directed to corresponding author, Dr. EV.

## References

[1] Franck-Larsson K Graf W Rönnblom A. Lower gastrointestinal symptoms and quality of life in patients with systemic sclerosis: a population-based study. Eur J Gastroenterol Hepatol. (2009) 21:176–82. doi: 10.1097/MEG.0b013e32831dac75, PMID: 19212206

[2] Good SD Lee JY Johnson RE Volkmann ER. A scoping review of the epidemiology of systemic sclerosis and its organ manifestations: 2018–2024. Curr Opin Rheumatol. (2025) 37:103. doi: 10.1097/BOR.0000000000001063, PMID: 39470126 PMC11779589

[3] Miller JB Gandhi N Clarke J McMahan Z. Gastrointestinal involvement in systemic sclerosis. J Clin Rheumatol. (2018) 24:328–37. doi: 10.1097/RHU.0000000000000626, PMID: 29095721 PMC6110377

[4] Omair MA Lee P. Effect of gastrointestinal manifestations on quality of life in 87 consecutive patients with systemic sclerosis. J Rheumatol. (2012) 39:992–6. doi: 10.3899/jrheum.110826, PMID: 22467930

[5] Nietert PJ Mitchell HC Bolster MB Curran MY Tilley BC Silver RM. Correlates of depression, including overall and gastrointestinal functional status, among patients with systemic sclerosis. J Rheumatol. (2005) 32:51–7., PMID: 15630725

[6] Andréasson K Lee SM Lagishetty V Wu M Howlett N English J. Disease features and gastrointestinal microbial composition in patients with systemic sclerosis from two independent cohorts. ACR Open Rheumatol. (2022) 4:417–25. doi: 10.1002/acr2.11387, PMID: 35174673 PMC9096523

[7] Andréasson K Alrawi Z Persson A Jönsson G Marsal J. Intestinal dysbiosis is common in systemic sclerosis and associated with gastrointestinal and extraintestinal features of disease. Arthritis Res Ther. (2016) 18:278. doi: 10.1186/s13075-016-1182-z, PMID: 27894337 PMC5126986

[8] Hevia A Milani C López P Cuervo A Arboleya S Duranti S. Intestinal dysbiosis associated with systemic lupus erythematosus. mBio. (2014) 5:e01548–01514. doi: 10.1128/mBio.01548-14, PMID: 25271284 PMC4196225

[9] Sokol H Seksik P. The intestinal microbiota in inflammatory bowel diseases: time to connect with the host. Curr Opin Gastroenterol. (2010) 26:327. doi: 10.1097/MOG.0b013e328339536b, PMID: 20445446

[10] Li Q Chang Y Zhang K Chen H Tao S Zhang Z. Implication of the gut microbiome composition of type 2 diabetic patients from northern China. Sci Rep. (2020) 10:5450. doi: 10.1038/s41598-020-62224-3, PMID: 32214153 PMC7096501

[11] Kulkarni P Devkumar P Chattopadhyay I. Could dysbiosis of inflammatory and anti-inflammatory gut bacteria have an implications in the development of type 2 diabetes? A pilot investigation. BMC Res. (2021) 14:52. doi: 10.1186/s13104-021-05466-2, PMID: 33549142 PMC7868023

[12] Zechner EL. Inflammatory disease caused by intestinal pathobionts. Curr Opin Microbiol. (2017) 35:64–9. doi: 10.1016/j.mib.2017.01.011, PMID: 28189956

[13] Clemente JC Manasson J Scher JU. The role of the gut microbiome in systemic inflammatory disease. BMJ. (2018) 360:j5145. doi: 10.1136/bmj.j5145, PMID: 29311119 PMC6889978

[14] David LA Maurice CF Carmody RN Gootenberg DB Button JE Wolfe BE. Diet rapidly and reproducibly alters the human gut microbiome. Nature. (2014) 505:559–63. doi: 10.1038/nature12820, PMID: 24336217 PMC3957428

[15] Kolodziejczyk AA Zheng D Elinav E. Diet–microbiota interactions and personalized nutrition. Nat Rev Microbiol. (2019) 17:742–53. doi: 10.1038/s41579-019-0256-8, PMID: 31541197

[16] . Dietary guidelines for the brasilian population. Ms (2015). Available online at: https://bvsms.saude.gov.br/bvs/publicacoes/dietary_guidelines_brazilian_population.pdf.

[17] Atzeni A Martínez MÁ Babio N Konstanti P Tinahones FJ Vioque J. Association between ultra-processed food consumption and gut microbiota in senior subjects with overweight/obesity and metabolic syndrome. Front Nutr. (2022) 9:976547. doi: 10.3389/fnut.2022.976547, PMID: 36299993 PMC9589409

[18] Cuevas-Sierra A Milagro FI Aranaz P Martínez JA Riezu-Boj JI. Gut microbiota differences according to ultra-processed food consumption in a spanish population. Nutrients. (2021) 13:2710. doi: 10.3390/nu13082710, PMID: 34444870 PMC8398738

[19] Lopes Cortes M Andrade Louzado J Galvão Oliveira M Moraes Bezerra V Mistro S Souto Medeiros D. Unhealthy food and psychological stress: the association between ultra-processed food consumption and perceived stress in working-class young adults. Int J Environ Res Public Health. (2021) 18:3863. doi: 10.3390/ijerph18083863, PMID: 33917015 PMC8103503

[20] Canhada SL Vigo Á Luft VC Levy RB Alvim Matos SM del Carmen Molina M. Ultra-processed food consumption and increased risk of metabolic syndrome in adults: the ELSA-brasil. Diabetes Care. (2023) 46:369–76. doi: 10.2337/dc22-1505, PMID: 36516280 PMC9887627

[21] Elizabeth L MaChado P Zinöcker M Baker P Lawrence M. Ultra-processed foods and health outcomes: A narrative review. Nutrients. (2020) 12:1955. doi: 10.3390/nu12071955, PMID: 32630022 PMC7399967

[22] Dai S Wellens J Yang N Li D Wang J Wang L. Ultra-processed foods and human health: An umbrella review and updated meta-analyses of observational evidence. Clin Nutr Edinb Scotl. (2024) 43:1386–94. doi: 10.1016/j.clnu.2024.04.016, PMID: 38688162

[23] Narula N Wong ECL Dehghan M Mente A Rangarajan S Lanas F. Association of ultra-processed food intake with risk of inflammatory bowel disease: prospective cohort study. BMJ. (2021) 374:n1554. doi: 10.1136/bmj.n1554, PMID: 34261638 PMC8279036

[24] van den Hoogen F Khanna D Fransen J Johnson SR Baron M Tyndall A. Classification criteria for systemic sclerosis: an ACR-EULAR collaborative initiative. Arthritis Rheumatol. (2013) 65:2737–47. 10.1002/art.38098 PMC3930146 24122180

[25] Tong M Jacobs JP McHardy IH Braun J. Sampling of intestinal microbiota and targeted amplification of bacterial 16S rRNA genes for microbial ecologic analysis. Curr Protoc Immunol. (2014) 107:Unit–7.41. Ed John E Coligan Al. doi: 10.1002/0471142735.im0741s107, PMID: 25367129 PMC4457454

[26] Blanco-Míguez A Beghini F Cumbo F McIver LJ Thompson KN Zolfo M. Extending and improving metagenomic taxonomic profiling with uncharacterized species using MetaPhlAn 4. Nat Biotechnol. (2023) 41:1633–44. doi: 10.1038/s41587-023-01688-w, PMID: 36823356 PMC10635831

[27] Subar AF Thompson FE Kipnis V Midthune D Hurwitz P McNutt S. Comparative validation of the Block, Willett, and National Cancer Institute food frequency questionnaires: the Eating at America’s Table Study. Am J Epidemiol. (2001) 154:1089–99. doi: 10.1093/aje/154.12.1089, PMID: 11744511

[28] National Cancer Institute, Epidemiology and Genomics Research Program. DHQ nutrient database. Available online at: https://epi.grants.cancer.gov/dhq2/database/.

[29] Monteiro CA Cannon G Moubarac JC Levy RB Louzada MLC Jaime PC. The UN Decade of Nutrition, the NOVA food classification and the trouble with ultra-processing. Public Health Nutr. (2018) 21:5–17. doi: 10.1017/S1368980017000234, PMID: 28322183 PMC10261019

[30] Khandpur N Rossato S Drouin-Chartier JP Du M Steele EM Sampson L. Categorising ultra-processed foods in large-scale cohort studies: evidence from the Nurses’ Health Studies, the Health Professionals Follow-up Study, and the Growing Up Today Study. J Nutr Sci. (2021) 10:e77. doi: 10.1017/jns.2021.72, PMID: 34589209 PMC8453454

[31] Khanna D Hays RD Maranian P Seibold JR Impens A Mayes MD. Reliability and validity of UCLA scleroderma clinical trial consortium gastrointestinal tract (UCLA SCTC GIT 2.0) instrument. Arthritis Rheumatol. (2009) 61:1257–63. doi: 10.1002/art.24730, PMID: 19714600 PMC2767193

[32] Cohen J. Statistical Power Analysis for the Behavioral Sciences. 2nd. Hillsdale, MI, USA: Lawrence Erlbaum Associates (1988).

[33] Levy RL Langer SL Romano JM Labus J Walker LS Murphy TB. Cognitive mediators of treatment outcomes in pediatric functional abdominal pain. Clin J Pain. (2014) 30:1033–43. doi: 10.1097/AJP.0000000000000077, PMID: 24469611 PMC4110203

[34] Aziz-Zadeh L Mayer E Labus J Ringold S Jayashankar A Kilroy E. Relationships between tryptophan-related gut metabolites, brain activity, and autism symptomatology. Res Sq. (2024). doi: 10.21203/rs.3.rs-4559624/v1, PMID: 40229237 PMC11997199

[35] Hayes AF. Beyond baron and kenny: statistical mediation analysis in the new millennium. Commun Monogr. (2009) 76:408–20. doi: 10.1080/03637750903310360

[36] R Core Team. R: A Language and Environment for Statistical Computing. Vienna, Austria: R Foundation for Statistical Computing (2013). Available online at: http://www.R-project.org/ (Accessed September 3, 2025).

[37] Rosseel Y. lavaan: an R package for structural equation modeling. J Stat Software. (2012) 48:1–36. doi: 10.18637/jss.v048.i02

[38] MacKinnon DP Lockwood CM Williams J. Confidence limits for the indirect effect: distribution of the product and resampling methods. Multivar Behav Res. (2004) 39:99. doi: 10.1207/s15327906mbr3901_4, PMID: 20157642 PMC2821115

[39] Cheung S. semhelpinghands: Helper Functions for Structural Equation Modeling (2024). Available online at: https://sfcheung.github.io/semhelpinghands/ (Accessed September 3, 2025).

[40] Blanco-Rojo R Sandoval-Insausti H López-Garcia E Graciani A Ordovás JM Banegas JR. Consumption of ultra-processed foods and mortality: A national prospective cohort in Spain. Mayo Clin Proc. (2019) 94:2178–88. doi: 10.1016/j.mayocp.2019.03.035, PMID: 31623843

[41] Sen A Brazeau AS Deschênes S Melgar-Quiñonez HR Schmitz N. The role of ultra-processed food consumption and depression on type 2 diabetes incidence: a prospective community study in Quebec, Canada. Public Health Nutr. (2023) 26:2294–303. doi: 10.1017/S1368980022002373, PMID: 36329635 PMC10641613

[42] Rurgo S Marchili MR Spina G Roversi M Cirillo F Raucci U. Prevalence of rome IV pediatric diagnostic questionnaire-assessed disorder of gut–brain interaction, psychopathological comorbidities and consumption of ultra-processed food in pediatric anorexia nervosa. Nutrients. (2024) 16:817. doi: 10.3390/nu16060817, PMID: 38542728 PMC10975836

[43] Schnabel L Buscail C Sabate JM Bouchoucha M Kesse-Guyot E Allès B. Association between ultra-processed food consumption and functional gastrointestinal disorders: results from the french nutriNet-santé Cohort. Off J Am Coll Gastroenterol ACG. (2018) 113:1217. doi: 10.1038/s41395-018-0137-1, PMID: 29904158

[44] Raoul P Cintoni M Palombaro M Basso L Rinninella E Gasbarrini A. Food additives, a key environmental factor in the development of IBD through gut dysbiosis. Microorganisms. (2022) 10:167. doi: 10.3390/microorganisms10010167, PMID: 35056616 PMC8780106

[45] Volkmann ER Chang YL Barroso N Furst DE Clements PJ Gorn AH. Association of systemic sclerosis with a unique colonic microbial consortium. Arthritis Rheumatol Hoboken NJ. (2016) 68:1483–92. doi: 10.1002/art.39572, PMID: 26749064 PMC5561666

[46] Vemuri R Sylvia KE Klein SL Forster SC Plebanski M Eri R. The microgenderome revealed: sex differences in bidirectional interactions between the microbiota, hormones, immunity and disease susceptibility. Semin Immunopathol. (2019) 41:265–75. doi: 10.1007/s00281-018-0716-7, PMID: 30298433 PMC6500089

[47] Volkmann ER Hoffmann-Vold AM Chang YL Jacobs JP Tillisch K Mayer EA. Systemic sclerosis is associated with specific alterations in gastrointestinal microbiota in two independent cohorts. BMJ Open Gastroenterol. (2017) 4:e000134. doi: 10.1136/bmjgast-2017-000134, PMID: 28761687 PMC5508636

