# Supplementary material for: Ultra-processed food intake is associated with increased gastrointestinal tract symptoms and alterations in gut microbiota in patients with systemic sclerosis
Source: Front Immunol. 2025 Oct 9;16:1610360. doi: 10.3389/fimmu.2025.1610360 (PMC12546150; doi:10.3389/fimmu.2025.1610360)
Supplement: Supplementary file 1 [file Table1.docx]

| **Supplemental Table 1. Food items that were classified as ultra-processed (NOVA group 4) from the Diet History Questionnaire II (DHQ-2) and included in the calculation of participant ultra-processed food intake.** | |
| --- | --- |
| **Food items** | **Food items, continued** |
| Orange juice | Burgers, non-fast food |
| Fruit juice | Hot dogs |
| Fruit drinks | Baked ham |
| Chocolate milk | Gravy |
| Meal replacement drinks | Bacon |
| Soda | Sausage |
| Sports drinks | Fish sticks |
| Energy drinks | Tofu |
| Liquor | Pizza |
| Cold cereal | Crackers |
| Applesauce | Corn bread |
| Salad dressing | Biscuits |
| French fries | Potato chips |
| Catsup | Corn chips |
| Stuffing, dressing, dumplings | Popcorn |
| Chili | Pretzels |
| Mexican food | Energy bars |
| Pancakes | Frozen yogurt |
| Macaroni and cheese | Ice cream |
| Bagels | Cake |
| Sandwich bread | Cookies |
| Non-sandwich bread | Donuts |
| Jam, jelly | Sweet muffins |
| Cold cuts | Fruit crisps, cobblers |
| Deli ham | Chocolate |
| Non-poultry cold cuts | Candy |
| Hamburgers, fast food | Artificial sweeteners |

| **Supplemental Table 2. Ultra-processed food items from the Diet History Questionnaire II (DHQ-2) that were excluded from our calculation of participant ultra-processed food intake given ambiguity in estimating portion sizes.** |
| --- |
| Food items |
| Margarine |
| Fats / Dressings |
| Syrup |
| Cream cheese |
| Mayonnaise |
| Nuggets |
| Pie |
| Coffee |
| Salad dressing |
| Cheesecake |
| Chocolate, fudge, or butterscotch toppings |
| Chow mein |
| Milkshakes or ice cream sodas |
| Pudding or custard |
| Whipped cream, regular |
| Whipped cream, substitute |
| Jell-o |
